# Hyperosmolar environment and intestinal epithelial cells: impact on mitochondrial oxygen consumption, proliferation, and barrier function *in vitro*

**DOI:** 10.1038/s41598-019-47851-9

**Published:** 2019-08-06

**Authors:** Marta Grauso, Annaïg Lan, Mireille Andriamihaja, Frédéric Bouillaud, François Blachier

**Affiliations:** 1 UMR PNCA, AgroParisTech, INRA, Université Paris-Saclay, 75005 Paris, France; 2 Institut Cochin, INSERM U1016, CNRS UMR8104, Université Paris Descartes, 75014 Paris, France

**Keywords:** Gastrointestinal models, Energy metabolism, Gastrointestinal models

## Abstract

The aim of the present study was to elucidate the *in vitro* short-term (2-h) and longer-term (24-h) effects of hyperosmolar media (500 and 680 mOsm/L) on intestinal epithelial cells using the human colonocyte Caco-2 cell line model. We found that a hyperosmolar environment slowed down cell proliferation compared to normal osmolarity (336 mOsm/L) without inducing cell detachment or necrosis. This was associated with a transient reduction of cell mitochondrial oxygen consumption, increase in proton leak, and decrease in intracellular ATP content. The barrier function of Caco-2 monolayers was also transiently affected since increased paracellular apical-to-basal permeability and modified electrolyte permeability were measured, allowing partial equilibration of the trans-epithelial osmotic difference. In addition, hyperosmotic stress induced secretion of the pro-inflammatory cytokine IL-8. By measuring expression of genes involved in energy metabolism, tight junction forming, electrolyte permeability and intracellular signaling, different response patterns to hyperosmotic stress occurred depending on its intensity and duration. These data highlight the potential impact of increased luminal osmolarity on the intestinal epithelium renewal and barrier function and point out some cellular adaptive capacities towards luminal hyperosmolar environment.

## Introduction

The composition of colonic luminal content may vary over the time. Indeed in healthy humans, diet can modify colonic microbiota composition and its metabolic activity^1^ with consequent changes of the bacterial metabolite concentrations, pH or osmolarity in the luminal content. Recent works have shown that several bacterial metabolites generated from undigested or not fully digested dietary and endogenous compounds, may affect the renewal of the colonic epithelium, its mitochondrial activity and/or its barrier function either in a beneficial or deleterious way depending on their luminal concentrations^2^. Little is known however on the consequences of an increase in luminal content osmolarity on the intestinal epithelium metabolism and functions, although luminal hyperosmolarity is observed in both physiological^3^ and pathological conditions in intestine, notably in inflammatory bowel diseases (IBD) where higher osmolarity has been measured in fecal fluid obtained from Crohn’s disease patients in comparison to healthy individuals^4–6^. Interestingly, we found in the rat model that consumption of a high-protein diet, that upraises the content of several amino acid-derived bacterial metabolites in large intestine, was concomitant with an augmented water retention in the large intestine luminal content, thus limiting bacterial metabolite concentration increase and hence luminal osmolarity due to the consumption of such diet^7,8^.

After initial cell shrinkage in response to hyperosmotic environnement, the cells increase the intracellular concentrations in inorganic ions and organic osmolytes. The aim of these changes is presumably to maintain water intracellular volume in a range compatible with normal cell functions, thus recovering a normal cell volume.

Mechanisms allowing cells to adapt to osmotic stress notably involve the nuclear factor of activated T cell 5 (NF-AT5), also known as tonicity-responsive enhancer-binding protein, targeting tonicity-responsive gene transcription^9,10^. This member of the NF-ĸB/rel family, is responsible for the up-regulation of a number of genes that elicit not only the osmoadaptive cell response but also influence a wider spectrum of biological processes^11^, of which some might be involved during IBD^12^. Indeed, hyperosmotic exposure of the epithelial cells resulted in the production of pro-inflammatory cytokines^13–15^. In addition to the activation of the transcription factor NF-AT5, studies on human intestinal epithelial cell (IEC) lines showed that hyperosmolarity stimulates the inflammatory cascade, a process that involves activation of MAP kinases, Na^+^/H^+^ exchangers (NHEs), as well as NF-ĸB, and results in IL-8 production^16^. Moreover, hypertonic solutions applied to the human colonic cell line model Caco-2 increases within few hours the inflammatory marker cyclooxygenase 2 (COX-2)^17^ whose gene contains NFAT-responsive element^18^. Furthermore in *in vitro* as well as *in vivo* experiments, a hyperosmotic environment raises the expression of the Ste20-like proline/alanine-rich kinase (SPAK) in colonocytes, which expression is associated with an increased epithelial permeability and intestinal inflammation^19,20^. It has been reported that osmotic stress induces epithelial barrier function disturbances both in Caco-2 cells and in mouse ileum, and demonstrated the implication of c-Jun NH_2_-terminal kinase-2 (JNK2) signaling pathway on tight junction (TJ) protein redistribution^21^. In addition, luminal hyperomolarity appears to be involved in the induction of inflammation by dextran sodium sulfate (DSS) in mice^22^ most probably by a calcium-mediated oxidative stress^23^, as reported for the JNK2-mediated osmotic stress-induced TJ disruption^24^.

In sum, it appears that a hyperosmotic environment may represent an environmental parameter which would favor colonic inflammation in predisposed individuals^25^.

In this context, and in order to improve the knowledge about the consequences of hyperosmolarity on intestinal epithelium metabolism and functions, the aim of this work was to measure the short-term (2-h) and longer-term (24-h) effects of an intermediate (500 mOsm/L) and a high (680 mOsm/L) hypertonic media on colonic epithelial cell viability, proliferation, energy metabolism, barrier and permeability functions in relationship with the expression of genes involved in these cellular characteristics. We used the Caco-2 epithelial cell line, which although presenting limitation for the study of intestinal drug absorption^26^, represents a well-established *in vitro* model useful for a first approach study of the effects of changing luminal environment on the colonic epithelium metabolism and barrier function. We tested on this model media rendered hyperosmotic by mean of the addition of the osmoactive sugar mannitol, this latter compound being poorly absorbed by the intestinal cells^27^.

## Results

### Hyperosmotic media decrease human colonocyte proliferation, mitochondrial activity and ATP cell content with no impact on their adherence and viability

**Figure 1:**
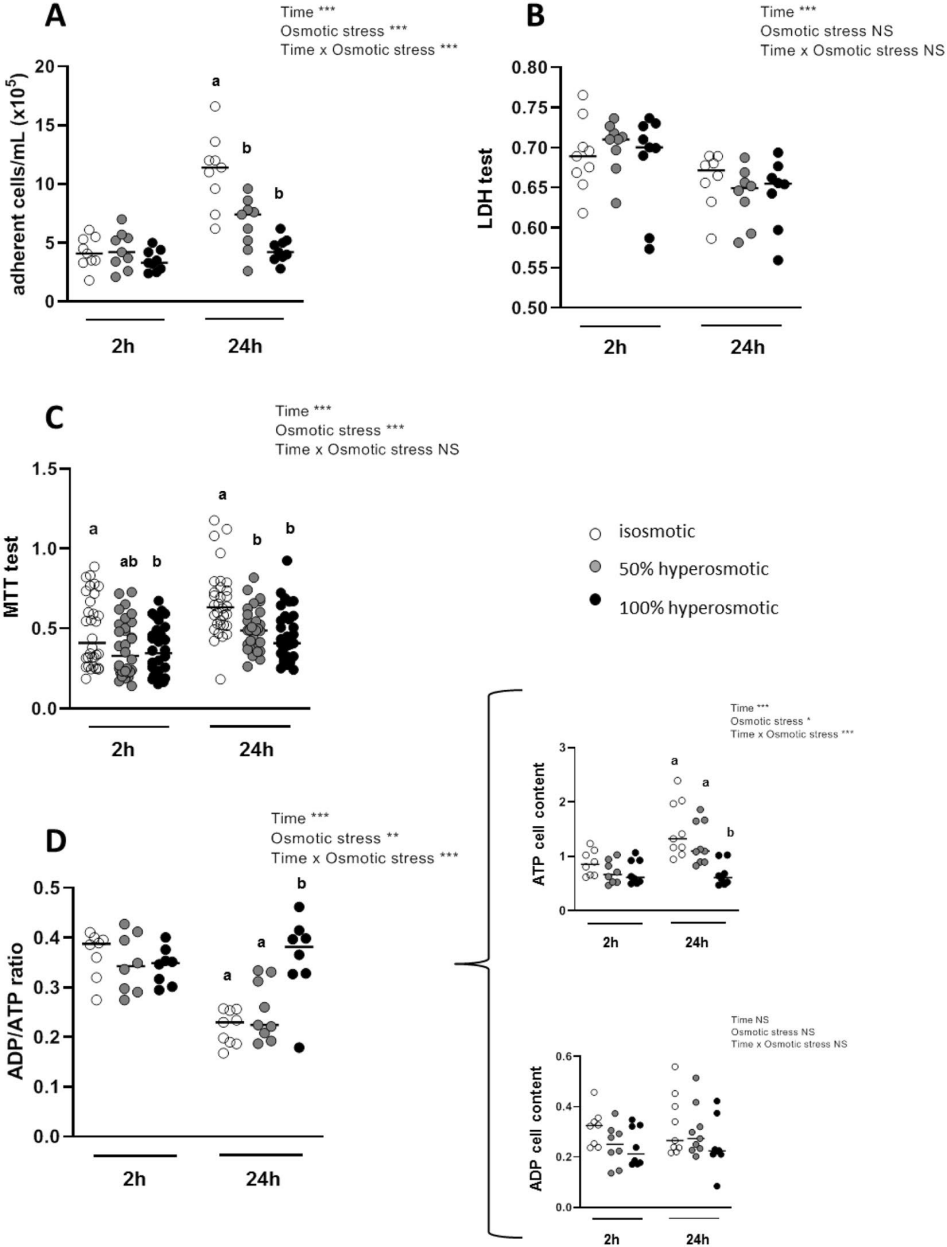
Effects of apical hyperosmolarity on cell viability. Assays were performed after treatment during 2- or 24-h with hyperosmotic media or isosmotic control medium. (**A**) Number of adhering cells measured by cell counting. (**B**) Cell viability estimated by measuring LDH released in the medium before and after 1% triton treatment and expressed as ratio of alive and total cells. (**C**) Cell viability evaluated with MTT test, and (**D**) ADP/ATP cell ratio from the amount of ATP and ADP cell content (expressed as relative luminescence units in the curly bracket). In (**A**) and (**B**) experiments, cells were plated on 24-well plates and grown for 3 days before hyperosmotic stress assay. In (**C**) and (**D**) experiments, cells were plated on 96-well plates and grown 3 days before hyperosmotic stress assay. Values are from three to four independent experiments (n = 8–32 for each experimental group). Mean significant differences (*P* < 0.05) are indicated by a different letter. Main factor and interaction effects are indicated with *P < 0.05, **P < 0.01 and ***P < 0.001. NS: Non-significant difference.

Hyperosmotic medium (both moderate and high, at 50% and 100% over isosmotic condition respectively), when tested during 2-h on undifferentiated Caco-2 cells, did not modify the number of adhering cells when compared to normal osmotic condition (Fig. 1A). However after 24-h treatment with hyperosmotic media, the mean number of adhering cells was much lower than in control, showing 40% and 60% decreases for 50% and 100% hyperosmotic media respectively (*P*-value < 0.001). Both the duration and the intensity of the osmotic stress, as well as their interaction, were involved in the effects on cell proliferation (Fig. 1A). The hyperosmotic media had no effect on cell viability as evaluated by measurement of the lactate dehydrogenase (LDH) activity released in the culture media (Fig. 1B). In good accordance with this latter result, the treatment of human colonocytes with the hyperosmotic media did not lead to an increase in the number of floating cells in the culture media after either 2- or 24-h from the onset of treatments (data not shown).

The methyltetrazoleum (MTT) reduction test, which reflects the mitochondrial activity in cells, showed a lower activity after 2-h in each hyperosmolar medium, such decrease being significant only after treatment with the 100% hyperosmolar medium (*P*-value = 0.049; Fig. 1C). However, a more pronounced decrease in mitochondrial activity after 24-h treatment was recorded, with an average of 26-to-30% lower MTT reduction to formazan in each hyperosmolar media when compared to the isosmotic control (*P*-value = 0.0016 et 0.0001 for 50% and 100% hyperosmotic stress respectively; Fig. 1C). Furthermore, the 2-h incubation of Caco-2 cells with each hyperosmotic media did not change the cell ADP/ATP ratio (Fig. 1D) when compared to the isosmotic condition. However, after 24-h incubation in the 100% hyperosmotic medium, ADP/ATP ratio increased (*P*-value < 0.0001) as a consequence of ATP cell content reduction (*P*-value < 0.0001; insert of Fig. 1D). This effect was dependent on both duration and osmotic stress intensity.

### Hyperosmotic media at the apical side affect the trans-epithelial electrical resistance, the barrier function and the morphology of human colonocyte monolayer

**Figure 2:**
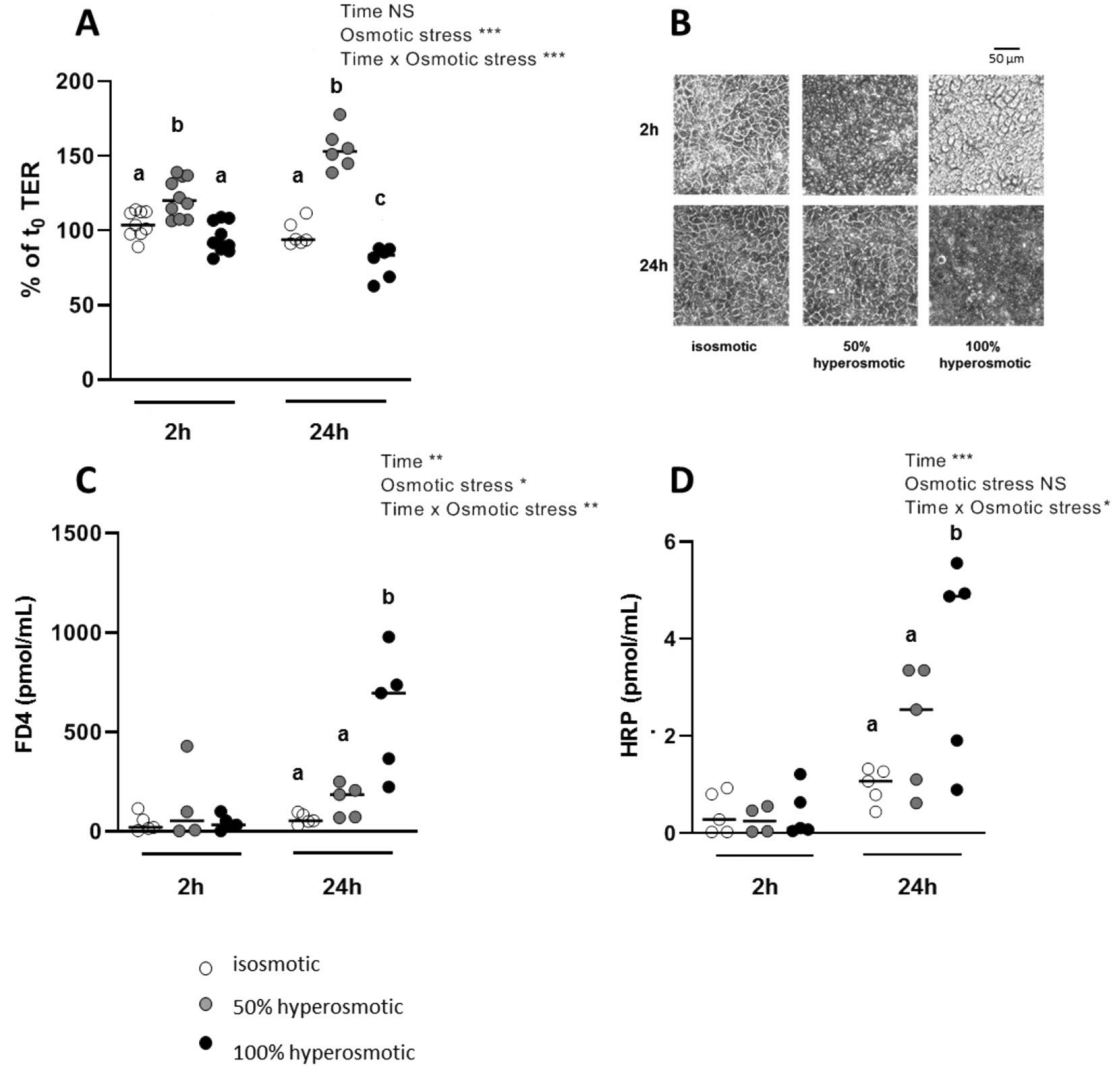
Effects of apical hyperosmolarity on trans-epithelial electrical resistance, cell morphology and epithelial permeability to macromolecules markers. Differentiated Caco-2 cells cultured on Tranwell filters for 15 days were treated during 2- or 24-h with hyperosmotic or control isosmotic media applied at the cellular apical side. (**A**) TER was measured before the hyperosmotic stress (t_0_) and after 2- or 24-h treatment. (**B**) Light microscope images of cell monolayers were acquired in the different experimental conditions using a 20x objective and the default exposition parameters. (**C**) Paracellular and (**D**) transcellular permeabilities were estimated by measuring the FD4 and HRP content respectively in the basal side medium at the end of the experiments. Values are from three independent experiments (n = 4–9 for each experimental group). Mean significant differences (*P* < 0.05) are indicated by a different letter. Main factor and interaction effects are indicated with *P < 0.05, **P < 0.01 and ***P < 0.001. NS: Non-significant difference.

Epithelial barrier function was assessed in Caco-2 cells by measuring the trans-epithelial resistance (TER) which is an indicator of epithelial integrity. Differentiated Caco-2 cells displayed a TER averaging 400.5 ± 6.5 ohm × cm^2^ (n = 35) at the onset of experiments. The TER was found stable after 2- or 24-h in isosmolar apical-basal media since it averaged respectively 104.4 ± 2.9% (n = 9) and 97.7 ± 3.3% (n = 6) of the initial TER. After apical hyperosmotic stress, the TER was markedly affected with opposite effects depending on hyperosmolar stress severity (Fig. 2A). In fact, in presence of 50% apical hyperosmolarity, the monolayer mean TER increased significantly to 122.0 ± 4.1% and 154.8 ± 5.6% of the initial value after 2- or 24-h respectively (*P*-value = 0.007 and <0.001; Fig. 2A). After 2-h of apical hyperosmotic stress, the cell boundaries observed under light microscope (Fig. 2B), appeared shrunk likely reflecting a monolayer lateral intercellular space (LIS) collapse after loss of sodium and water. However, after 24-h in 50% apical hyperomotic medium, cell morphology appeared similar to isosmotic control cells. At 2-h the apical 100% hyperosmolar media did not change the Caco-2 monolayer TER with respect to isosmolar condition as it did after 24-h with an average TER decrease to 79.0 ± 4.3% of the control value respectively (*P*-value = 0.04; Fig. 2A). However, optical microscopic observation of cell monolayer after 2-h from the onset of treatment with 100% hyperosmotic stress showed some bourgeoning cells that evolved in 24-h in monolayer disorganization with cell membrane boundaries being no longer discernible (Fig. 2B). In that case, it is likely that a huge water or electrolyte conductance impacted the overall measured TER.

Concentration of the paracellular permeability marker fluorescein isothiocyanate (FITC)-dextran applied at the apical side of differentiated Caco-2 cells increased at the basal side after 24-h apical incubation with the 100% hyperosmotic medium (*P*-value < 0.001; Fig. 2C), in accordance with the TER dropping after 24-h. Using the antigen-sized protein horseradish peroxidase (HRP) as a marker for the macromolecule transcellular transport through the Caco-2 cell monolayer, an effective apical-to-basal flow of this molecule was found only after 24-h with 100% hyperosmotic stress (*P*-value = 0.004; Fig. 2D).

In order to know if differenciated intestinal cells raise their permeability to macromolecules and water to equilibrate the apical hyperosmolarity, osmolarity of apical and basal media was measured after 2- and 24-h from the onset of the hyperosmotic stresses. We found that the 50% hyperosmotic apical medium moved to 38 ± 1.9% (n = 6) and to 16.7 ± 0.7% (n = 6) of the initial osmolarity after 2- and 24-h respectively. Similarly, the 100% hyperosmotic apical medium moved to 74 ± 1.1% (n = 6) and to 34.7 ± 0.3% (n = 6) of the initial osmolarity after 2- and 24-h respectively, thus in good accordance with our hypothesis.

To characterize the differenciated Caco-2 cell electrolyte permeability under apical hyperosmotic condition, we measured the monolayer trans-epithelial potential (*V*_t_) and short-circuit current (*I*_sc_) with an Ussing chamber apparatus. In Caco-2 cell monolayers incubated with the control isosmotic medium, we observed a *V*_t_, of −1.5 ± 0.6 mV (negative apical side) and a *I*_sc_ of 4.9 ± 2.9 µA/cm^2^. After incubation for 24-h with 50% hyperosmotic medium applied at the apical side of cell monolayers, both *V*_t_ and *I*_sc_ inversed their polarity to 1.4 ± 0.2 mV (n = 3) and −3.1 ± 1.6 µA/cm^2^ (n = 3) respectively. After incubation for 24-hours with the highly hyperosmotic medium, the *V*_t_ and the *I*_sc_ were almost equal to zero, averaging 0.5 ± 0.3 mV (n = 3) and −0.8 ± 0.9 µA/cm^2^ (n = 3) respectively.

### Hyperosmotic media affect oxygen consumption in human colonocytes

**Figure 3:**
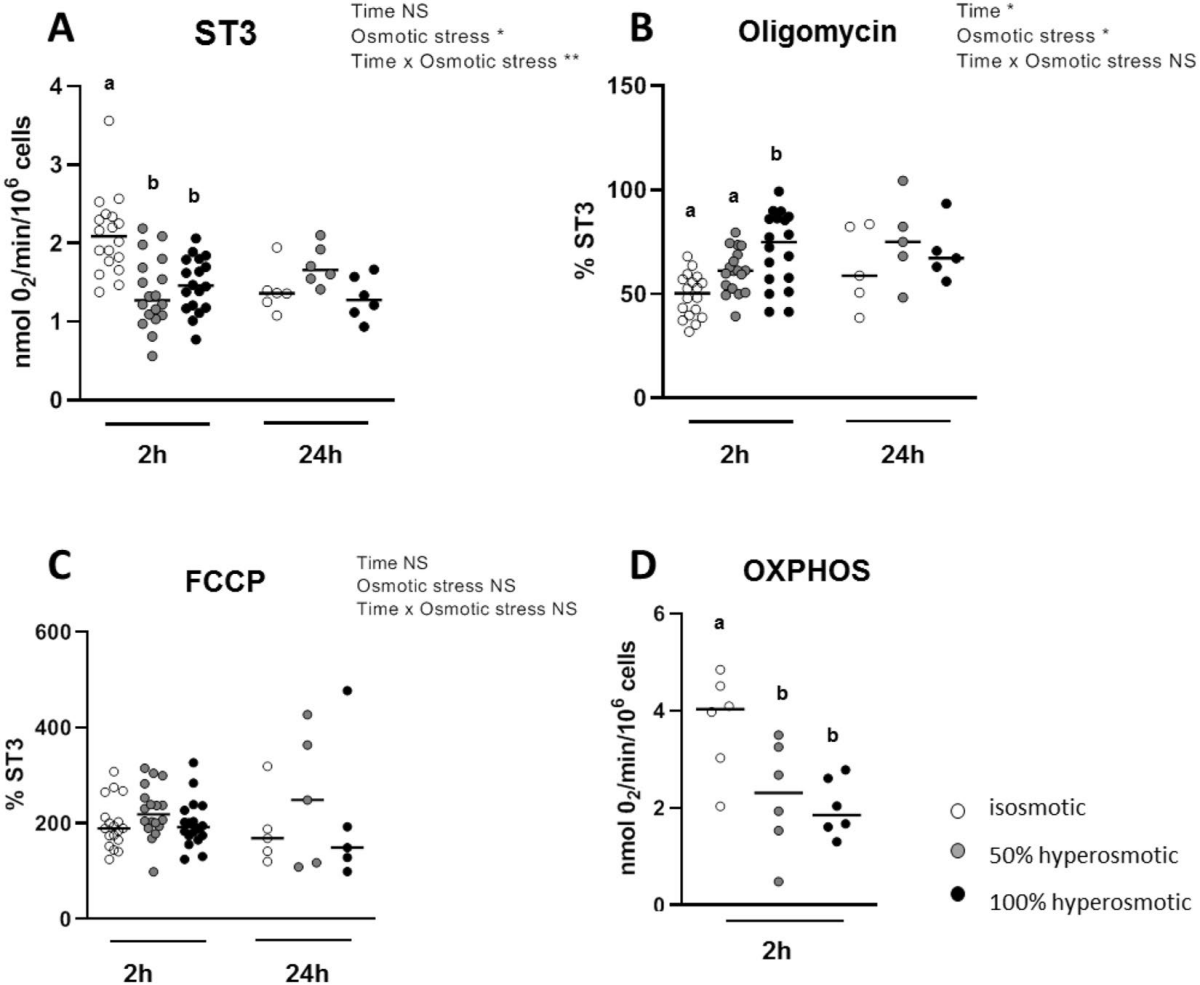
Effects of hyperosmolarity on Caco-2 cells oxygen consumption. Seven days after seeding, cells were cultured in isosmotic or hyperosomotic (50% and 100%) culture media during 2- or 24-h and then isolated and used for basal oxygen (ST3) consumption measurement (**A**) without any exogenous agent, (**B**) after addition of the Fo/F1 ATPase inhibitor oligomycin and (**C**) after addition of the uncoupler FCCP. OXPHOS was calculated for the 2-h hyperosmotic stress and corresponds to the oxygen consumption rate measured in permeabilized cells in presence of 1.5 mM saturated ADP concentration (**D**). Values are from three independent experiments (n = 5–18 for each experimental group). Mean significant differences (*P* < 0.05) are indicated by a different letter. Main factor and interaction effects are indicated with *P < 0.05, **P < 0.01 and ***P < 0.001. NS: Non-significant difference.

When Caco-2 cells were exposed to hyperosmolar media for 2-h, a significant reduction in basal oxygen consumption (ST3) was observed when compared to isosmolar group. Basal oxygen consumption (2.1 ± 0.1 nmol O_2_/min/10^6^ cells, n = 18) was reduced to 1.4 ± 0.1 nmol O_2_/min/10^6^ cells (n = 18) and 1.5 ± 0.1 nmol O_2_/min/10^6^ cells (n = 18) for 50% and 100% hyperosmotic stress (*P*-value < 0.0001 and = 0.0001 respectively; Fig. 3A). This decrease was transient since it was not observed after 24-h cell treatment. Oxygen consumption percentage relative to ST3 measured in presence of oligomycin, that measures the mitochondrial proton leak which favors uncoupling between oxygen consumption and ATP synthesis, was augmented after 2-h from 49.3 ± 2.4% (n = 18) to 61.0 ± 2.5% of ST3 (n = 18) and to 71.4 ± 4.3% of ST3 (n = 18) with 50% and 100% hyperosmolar treatment respectively with only the latter being a significant increase (*P*-value < 0.0001; Fig. 3B). However, such increases were not measured after 24-h. The hyperosmolar media had no effects on the oxygen consumption measured in presence of the uncoupler carbonyl cyanide 4-(trifluoromethoxy) phenylhydrazone (FCCP), meaning that the maximum respiratory capacity of cells remained unchanged (Fig. 3C). Using permeabilized human colonocytes, our results showed that oxidative phosphorylation (OXPHOS) lowered significantly in cells treated during 2-h with the 50% or 100% hyperosmolar media when compared to control medium (*P*-value = 0.04 and 0.02 respectively; Fig. 3D).

### Hyperosmotic media affect expression of genes linked to mitochondrial energy metabolism, tight junction proteins, and water and electrolyte transporters in association with increased interleukin-8 expression and secretion

Due to the inhibitory effects of the hyperosmolar media on mitochondrial oxygen consumption seen after 2-h treatement, we studied the consequences of an increase in medium osmolarity on expression of several genes encoding mitochondrial proteins involved in oxidative phosphorylation and in citric acid cycle both in growing cells recovered 7 days after seeding and in monolayers of differentiated Caco-2 cells (Table 1). The genes studied encode the following mitochondrial proteins: the NADH-ubiquinone oxidoreductase 75 kDa subunit (*NDUFS1* gene); the succinate dehydrogenase complex subunit D (*SDHD* gene) that links two important pathways in energy conversion, the Kreb’s cycle and oxidative phosphorylation; the cytochrome b-c1 complex subunit 7 (*UQCRB* gene), that participates in the transfer of electrons after binding of ubiquinone; the cytochrome c oxidase subunit 5B (*COX5B* gene) that is the terminal enzyme of the mitochondrial respiratory chain and belongs to a multi-subunit enzyme complex that couples electron transfer from cytochrome c to molecular oxygen contributing to a proton electrochemical gradient across the inner mitochondrial membrane; the ATP synthase subunit beta (*ATP5F1B* gene) that catalyzes ATP synthesis by utilizing the electrochemical gradient of protons across the inner membrane during oxidative phosphorylation; and the citrate synthase (*CS* gene) that is a Krebs’s enzyme catalyzing the synthesis of citrate from oxaloacetate and acetyl coenzyme A. After short-term hyperosmotic stress (2-h), none of the tested genes were affected in their expression when compared with isosmotic control both in growing cells and differentiated monolayer (data not shown). However, after 24-h from the treatment onset on proliferative Caco-2, *NDUFS1*, *SDHD* and *UQCRB* expression increased after cell incubation with 100% hyperosmolarity medium (*P*-value < 0.001, =0.002 and <0.001, respectively). In differentiated cells, with both intermediate and high hyperosmotic media only *NDUFS1* and *ATP5F1B* expression were significantly increased when compared to the isosmotic condition (*P*-value = 0.001 and 0.01 respectively in 50% hyperosmotic and = 0.006 and 0.01 respectively in 100% hyperosmotic).

**Table 1:** Effects of apical hyperosmolarity on expression of genes encoding for proteins involved in mitochondrial energy metabolism in Caco-2 monolayer.

24-h stress on proliferative cells				24-h stress on differentiated cells			Statistical effects		
*Gene*	iso	50% hyper	100% hyper	iso	50% hyper	100% hyper	Stage	Osmotic	Stage x Osmotic
*NDUFS1*	1.00 ± 0.04 (a)	0.92 ± 0.02 (a)	1.45 ± 0.08 (b)	1.00 ± 0.03 (a)	1.28 ± 0.04 (b)	1.24 ± 0.05 (b)	NS	***	***
*SDHD*	1.08 ± 0.19 (a)	1.03 ± 0.10 (a)	1.71 ± 0.14 (b)	1.01 ± 0.05	1.26 ± 0.06	1.33 ± 0.12	NS	***	NS
*UQCRB*	1.01 ± 0.05 (a)	0.85 ± 0.02 (a)	1.42 ± 0.07 (b)	1.00 ± 0.04	1.15 ± 0.02	1.17 ± 0.04	NS	***	***
*COX5B*	1.01 ± 0.07	0.95 ± 0.04	1.04 ± 0.02	1.00 ± 0.03	1.07 ± 0.05	1.13 ± 0.02	NS	NS	NS
*ATP5F1B*	1.02 ± 0.09	0.88 ± 0.05	0.89 ± 0.05	1.00 ± 0.04 (a)	1.32 ± 0.08 (b)	1.31 ± 0.06 (b)	***	NS	***
*CS*	1.00 ± 0.03	1.28 ± 0.04	1.24 ± 0.05	1.00 ± 0.04	1.08 ± 0.05	1.05 ± 0.03	*	NS	*

Relative gene expressions were measured in proliferative (7 days after seeding) and differentiated (15 days after seeding) Caco-2 cells after 24-h of osmotic treatments. Values are mean ± s.e.m. from three independent experiments each in duplicate (n = 6). Mean significant differences (*P* < 0.05) are indicated by a different letter. Main factor and interaction effects are indicated with *P < 0.05, **P < 0.01 and ***P < 0.001. NS: Non-significant difference.

Regarding genes encoding proteins involved in colonic epithelium barrier function and water and electrolyte movements, we restricted the study of their expression to differentiated Caco-2 monolayer for which TJ between neighboring cells are established (Table 2). When cells were incubated 2-h in presence of 50% apical hyperosmolar medium, expression of the genes encoding barrier-forming TJ proteins occludin (*OCLN*), claudin 1 (*CLDN1*) and claudin 5 (*CLDN5*), and the scaffolding TJ protein ZO-1 (*TJP1*) was augmented (*P*-value < 0.001, <0.001, =0.002 and =0.002 respectively), in accordance with the higher TER measured in that condition (Fig. 2A). However 100% hyperosmotic apical medium had the same effect on the *CLDN5* expression (*P*-value = 0.006). Interestingly, incubating the apical side with either 100% hyperosmolar medium, expression of the gene encoding permeability-promoting claudin 2 (*CLDN2*) was tremendously increased (*P*-value < 0.001). After 24-h from the hyperosmotic stress onset, all tested tight junction proteins recovered a gene expression similar to isosmotic control, with the exception of *JAMA*, the gene encoding the junction adhesion molecule (also known as F11 receptor, *F11R*, that localizes at cell-cell contacts adjacent to TJ proteins), that showed an higher expression in the 100% hyperosmotic condition compared to isosmotic control (*P*-value = 0.01).

**Table 2:** Effects of apical hyperosmolarity on expression of genes encoding tight junction, electrolytes transport and signaling proteins in Caco-2 monolayer.

2-h stress on differentiated cells				24-h stress on differentiated cells			Statistical effects		
Gene	iso	50% hyper	100% hyper	iso	50% hyper	100% hyper	Time	Osmotic	Time × Osmotic
**Tight junction proteins**									
*OCLN*	1.12 ± 0.21 (a)	2.79 ± 0.58 (b)	1.41 ± 0.26 (a)	1.01 ± 0.05	0.98 ± 0.04	1.16 ± 0.05	**	*	*
*TJP1*	1.10 ± 0.23 (a)	2.34 ± 1.07 (b)	1.07 ± 0.15 (a)	1.00 ± 0.04	0.77 ± 0.02	0.85 ± 0.06	**	*	**
*JAMA*	1.03 ± 0.11 (ab)	1.26 ± 0.13 (a)	0.85 ± 0.10 (b)	1.00 ± 0.03 (a)	1.21 ± 0.03 (ab)	1.45 ± 0.05 (b)	**	NS	***
*CLDN1*	1.02 ± 0.09 (a)	1.67 ± 0.13 (b)	1.29 ± 0.10 (a)	1.02 ± 0.08	1.05 ± 0.08	1.04 ± 0.05	***	*	**
*CLDN2*	1.26 ± 0.31 (a)	2.53 ± 0.31 (a)	10.59 ± 0.60 (b)	1.08 ± 0.17	1.77 ± 0.22	0.59 ± 0.13	***	***	***
*CLDN5*	0.99 ± 0.12 (a)	2.75 ± 0.48 (b)	2.62 ± 0.55 (b)	1.04 ± 0.13	0.66 ± 0.07	0.45 ± 0.05	***	NS	**
**Electrolytes transport proteins**									
*ATPA1A*	1.12 ± 0.25	1.18 ± 0.21	1.27 ± 0.25	1.00 ± 0.02	0.91 ± 0.03	0.81 ± 0.03	NS	NS	NS
*NHE1*	1.08 ± 0.19 (a)	2.51 ± 0.21 (b)	1.83 ± 0.18 (c)	1.01 ± 0.06	0.84 ± 0.03	1.03 ± 0.06	***	**	***
*NHE3*	1.30 ± 0.38	1.37 ± 0.35	1.10 ± 0.40	1.01 ± 0.05	0.82 ± 0.02	0.66 ± 0.02	NS	NS	NS
*NKCC1*	1.02 ± 0.10	1.38 ± 0.06	1.16 ± 0.13	1.02 ± 0.08 (a)	0.92 ± 0.07 (a)	2.59 ± 0.40 (b)	**	**	***
*AQP3*	1.02 ± 0.10 (a)	1.78 ± 0.26 (b)	1.27 ± 0.22 (b)	1.01 ± 0.08 (a)	0.56 ± 0.03 (b)	0.52 ± 0.04 (b)	***	NS	***
**Signaling proteins**									
*NFAT5*	1.01 ± 0.05 (a)	3.17 ± 0.27 (b)	2.12 ± 0.15 (c)	1.02 ± 0.09	0.89 ± 0.03	1.22 ± 0.06	***	***	***
*PTGS2*	1.03 ± 0.10(a)	4.73 ± 0.55 (b)	3.70 ± 0.18 (c)	1.02 ± 0.09	1.13 ± 0.08	0.93 ± 0.04	***	***	***
*IL-6*	1.16 ± 0.28 (a)	7.67 ± 1.57 (b)	9.30 ± 2.05 (b)	1.07 ± 0.17	0.92 ± 0.19	0.83 ± 0.11	***	**	**
*CXCL8*	1.25 ± 0.36 (a)	17.79 ± 1.95 (b)	5.96 ± 1.06 (c)	1.02 ± 0.09	1.02 ± 0.16	0.90 ± 0.13	***	***	***

Relative gene expressions were measured in differentiated (15 days after seeding) Caco-2 after 2-h or 24-h apical hyperosmotic stress. Values are mean ± s.e.m. from three independent experiments each in duplicate (n = 6). Mean significant differences (*P* < 0.05) are indicated by a different letter. Main factor and interaction effects are indicated with *P < 0.05, **P < 0.01 and ***P < 0.001. NS: Non-significant difference.

Regarding the expression of genes encoding for proteins involved in epithelium electrolytes and water permeability, expression of the genes encoding the Na^+^/K^+^ ATPase subunit alpha 1 (*ATP1A1*), the enzymatic subunit of the Na^+^/K^+^-ATPase pump (that generates the apical negative *V*_t_), was not affected after either a short-term or a 24-h apical hyperomotic stress. Only a 2-h apical incubation with both hyperosmolar media increased the expression of the Na^+^/H^+^ exchanger type 1 gene (*NHE1*). Indeed, an average 2.5-fold (*P*-value < 0.001) and 1.8-fold (*P*-value = 0.005) increase in *NHE1* expression was recorded for intermediate and high hyperosmolar media respectively, and at 24-h from the treatment onset the expression of *NHE1* recovered a level similar to isosmotic control. The expression of gene encoding the Na^+^/H^+^ exchanger type 3 (*NHE3*), an ion exchanger that specifically allows electroneutral sodium absorption at the luminal membrane of colonocytes, was not affected. The expression of gene encoding for the basolateral Na^+^-K^+^-Cl^−^ co-transporter (*NKCC1*), an ion transporter that is localized at the basolateral membrane of the crypt colonocytes where it takes up chloride anions, appeared increased only after 24-h of 100% apical hyperosmotic treatment with 2.5-fold isosmotic control average value (*P*-value < 0.001). The expression of gene coding for aquaporin 3 (*AQP3*), that in colonic mucosal epithelial cells is permeable to water and small solutes, was increased by short-term incubation in intermediate hyperosmolar medium (*P*-value = 0.007), but was reduced at 24-h of the both hyperosmotic treatment (*P*-values < 0,001).

**Figure 4:**
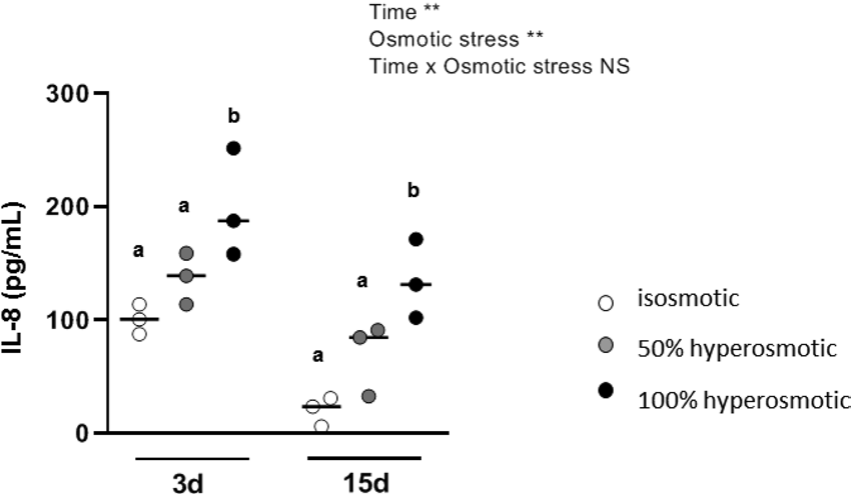
Delayed effects of apical hyperosmolar media on IL-8 secretion by Caco-2 cells. Secreted IL-8 after 24-h apical hyperosmotic stress was detected by ELISA test on culture media obtained from undifferentiated Caco-2 cells grown for 3 days, or from media recovered at the basal side of differentiated Caco-2 monolayer after 15 days. Values are from three independent experiments (n = 3 for each experimental group). Mean significant differences (*P* < 0.05) are indicated by a different letter. Main factor and interaction effects are indicated with *P < 0.05, **P < 0.01 and ***P < 0.001. NS: Non-significant difference.

Finally the expression of the gene encoding NF-AT5 (*NFAT5*) was increased 2-h after hyperosmotic stresses (*P*-value < 0.001 for both stresses). However, it returned back to basal expression level after 24-h. The same results were observed for expression of genes encoding for the pro-inflammatory COX-2 (*PTGS2*), interleukin-6 (*IL-6*) and interleukin-8 (*CXCL8*) (*P*-value < 0.001). We reasoned that the delay between the increased expression of *CXCL8* after 2-h treatment with hyperosmolar media, and the corresponding IL-8 protein increased production and accumulation in the culture medium measured after 24-h treatment would explain the results obtained (Fig. 4). Due to previously published studies showing that hyperosmotic stress increases pro-inflammatory cytokine IL-8 secretion^16^, we used this latter interleukin as a positive control for the effect of hyperosmotic stress. Such a latter increase in IL-8 secretion was statistically significant for both undifferentiated and differentiated Caco-2 cells after treatment with the higher hyperosmolar medium (*P*-value = 0.01 and 0.003 respectively).

## Discussion

The present study reports that hyperosmolar environment altered mitochondrial energy metabolism and cell proliferation without affecting IEC viability. Barrier function was also impacted but to a different extent according to the intensity of the osmotic stress and its duration.

Indeed, the results obtained in the present study clearly show that hyperosmotic stress was responsible for a rapid and transient mitochondrial dysfunction in regards to its inhibitory effect on the basal oxygen consumption. This dysfunction was concomitant with a rise in colonocyte oxygen consumption in the presence of oligomycin, at least in presence of a high hyperosmotic environnement, thus indicating an increased proton leak through the mitochondrial inner membrane and a decreased ATP production. The fact that in our experimental conditions, the maximal respiratory capacity, measured in presence of FCCP, was not modified when cells were exposed to the hyperosmolar medium indicates that the electron chain capacity was not limitant for the oxidative phosphorylation. Nevertheless the OXPHOS rate, measured after 2-h of hyperosmotic stress onset in permeabilized cells (i.e. conditions where ADP and substrates were not limitant) was also lower in cell treated with hyperosmolar media. This suggests that hyperosmolar media, in addition to their uncoupling effect, affects the ATP synthase activity. In that context, it is tempting to interpret the overall increased expression of genes linked to the mitochondrial energy metabolism after 24-h treatment with the hyperosmolar medium, as an adaptative process participating in the restoration of normal oxygen consumption. Despite this adaptation, the ATP content in colonocytes remained lower after 24-h in 100% hyperosmolar condition likely as a consequence of a decrease in the ATP production efficiency due to the uncoupling effect of the hyperosmolar medium, and presumably to a lower ATP synthase activity. It has been observed the same effects of hyperosmolar media in IMCD3 kidney cells and such effect on the mitochondrial activity was attributed to a decrease in the matrix volume secondary to the acute osmolarity increase in the extracellular medium, and hence in the cell cytoplasm^28^. The osmotic induction of the mitochondrial matrix volume reduction *in vitro* is considered to adversely affect the oxidation of respiratory substrates and ATP synthesis^29^.

In addition, a slowdown of cell proliferation in a context of impaired energy metabolism may be considered as a way to spare ATP^30^ since the synthesis of macromolecules that are necessary for cell mitosis is an ATP-consuming process^31^. Indeed, Buttgereit and Brand documented that when the cell respiration is inhibited by 30%, the rate of protein and polynucleotide synthesis is decreased by 40%^32^. However, viability was not affected by hyperosmolar conditions, like previously reported with a 600 mOsm/L hyperosmotic stress on Caco-2 cells^21^.

It was previously demonstrated that a hyperosmotic environment is able to induce a signal related to colonic cell survival, via the osmolarity regulator NF-AT5^33^. Among the early cell responses to hypertonicity, cell shrinkage leads to the nuclear import and accumulation of the transcription regulator NF-AT5 to elicit the osmoadaptive cell response^10,11,34^ consisting in a gene expression program that attempts to restore cell volume but also to favor cell proliferation and differentiation^35^. NF-AT5 nuclear dimerization also regulates cytokine gene transcription in response to osmotic stress^36^, and COX-2 expression is induced by NF-AT5 in renal epithelial cells, suggesting a cytoprotective role for COX-2^37^. Our findings strongly suggest that in colonocytes, the cell shrinkage observed after 2-h of apical hyperosmotic stress leads to a rapid nuclear accumulation of NF-AT5 allowing transient transcription of some pro-inflammatory (*IL6*, *CXCL8* and *PTGS2*) markers in response to luminal hyperosmolarity, whose concentrations in the incubation medium are increased later (e.g. IL-8 accumulation measured at 24-h). Such gene expression was found to return to the basal level when the apical to basal osmotic difference drops as observed 24-h after the stress onset.

Regarding the functional aspects of our study, our results confirm that hyperosmotic stress alters TER and increases macromolecule permeability indicating that epithelial barrier function was differently affected according to intensity of the osmotic stress. Indeed, in the medium grade apical hyperosmolarity condition, the TER increased after 2-h treatment when compared to control and presumably as a consequence of monolayer lateral intercellular space collapse^3,38^. After 24-h, the further TER increase recorded in this osmolarity condition may be due to an higher presence of TJ proteins *(OCLN, TJP1, CLDN1, CLDN5)*, related to the marked overexpression of related genes observed transiently after hyperosmotic stress. The duration of 50% hyperosmotic stress did not appear as a significant factor in generating the elevated TER seen at 2-h and 24-h respectively (Fig. 2A, P-value = 0.13). In contrast, using the 100% hyperosmolar medium, we found that TER was reduced significantly compared to control only after 24-h but not 2-h treatment. To explain this latter result, we propose that modification of water or electrolyte conductance after 2 h-treatment could explain the minor effect of hyperosmolarity on TER. This initial phase of TER modification was clearly detected after 24-h, at a time when the fall in TER and the observed poor monolayer morphology would be associated with TJ disruption. Higher paracellular permeability associated with TJ disruption *in vivo* has been previously associated with mucosal inflammatory process^39,40^.

This barrier impairment might be related to the high transient increase in the leaky claudin 2^41,42^ in Caco-2 monolayer. This TJ protein forms a paracellular water and cation-selective pore-channel^43,44^ that leads to the subsequent augmented TJ permeability^45^. In our experiments, this may provide a way for a transient basal-to-apical water flux in an attempt to reduce the apical hyperosmolarity. Instead, as detected after 24-h treatment, apical-to-basal macromolecule flux is likely strongly increased by loss of junctional integrity as a consequence of the lower expression of *TJP1* and *CLDN5*^37,46^. TJ integrity is known to be compromised by pro-inflammatory stimuli^47^ and, in good accordance with these results, we found in hyperosmotic stress conditions that Caco-2 cells transiently raised the expression of IL-6 and IL-8 genes, with an effective accumulation of IL-8 in the culture media after 24-h both in undifferentiated and differentiated cells submitted to the high hyperosmotic medium. Regarding the increased expression of gene encoding for the F11 receptor, *JAMA*, recorded after 24-h in hyperosmotic apical media, we interpret such a rise as part of the cell adaptive mechanism to the hyperosmotic environment on the basis of its known role in TJ integrity in epithelia. In other words, we propose that such an increase may represent a way to restore the cell morphology and the epithelial barrier integrity^48^.

The pro-inflammatory stimuli recorded in our study after the 24-h treatment with the hyperosmotic media may be responsible for the decreased expression of *AQP3*^49,50^. Indeed, aquaporin 3 decreased in the rat colon after a pro-inflammatory stimulation^51^.

In a healthy colonic epithelium, the activity of the Na^+^/K^+^-ATPase pump situated on the basolateral cell membrane extrudes sodium ions from the cell thus participating in the luminal negative *V*_t_ and generating the driving force necessary for the absorption of these ions from the apical side. The *I*_sc_ of the colonic epithelium, which is measured with the Ussing technique, is linked to the *V*_t_ reflecting all the ionic currents across the epithelium^38^. Therefore, both parameters describe the epithelial active transport capability, and our data indicate that under apical hyperosmotic environment, the ionic transport is rapidly perturbed. In fact depending on the severity of the hyperosmotic stress, the monolayer *V*_t_ was either inversed (with luminal side being positive) or nearly abolished, thus suggesting an inversion of the ATP-dependent sodium flux, or a suppression of the cell sodium driving force respectively. Significantly lower colonic mucosal *V*_t_, *I*_sc_ and TER, depending on defect in active sodium absorption, have been described in situation of active ulcerative colitis and Crohn’s disease^52^ with a reversal of the mucosal *V*_t_ and of sodium flux or of water flux respectively^53,54^. Mechanisms underlying these electrolytes disorders include reduced Na^+^/K^+^-ATPase activity^55^ rather than lower gene expression.

Epithelial NHEs exchange intracellular H^+^ for extracellular Na^+^ providing the electroneutral sodium absorption by secondary active transport and, in the meantime, contributing to maintain the intracellular pH^56^. NHE-1 isoform is localized at the basolateral membrane of colonocytes and intervenes in the cell volume regulation^57^. Thus, the early increased expression of the genes coding for NHE-1 and NKCC1 suggests an improved basal-to-apical water flow related to the chloride secretion. In contrast to NHE-1, NHE-3 is exclusively localized at the luminal membrane of colonocytes, where it largely contributes to electroneutral sodium absorption^58^. The expression of NHE-3 has been found downregulated by chronic inflammatory stimuli^59^, and the activity of NHE-3 is inhibited by hypertonic cell shrinkage thus leaving sodium and water unabsorbed at the luminal side of the epithelium^57^. However discontinued sodium and fluid absorption involving inhibition of NHE-3 activity and/or decreased expression of its corresponding gene have been described in inflammatory diseases and contribute to inflammatory diarrhea^60^. It appears that the complex modification of the expression of the genes encoding for these ion transporters reflects the adaptive mechanisms towards hyperosmotic stress. Overall, our results regarding the expression of genes encoding for electrolytes and water permeability suggest a diverse response pattern depending on the strength of the hyperosmolarity stress.

The complex interplay between the parameters measured and the implication of adaptive processes that are involved in our simplified model in response to an increase in extracellular osmolarity would certainly gain in clarity by performing a more complete kinetic experiment with intermediate time points.

## Conclusions

**Figure 5:**
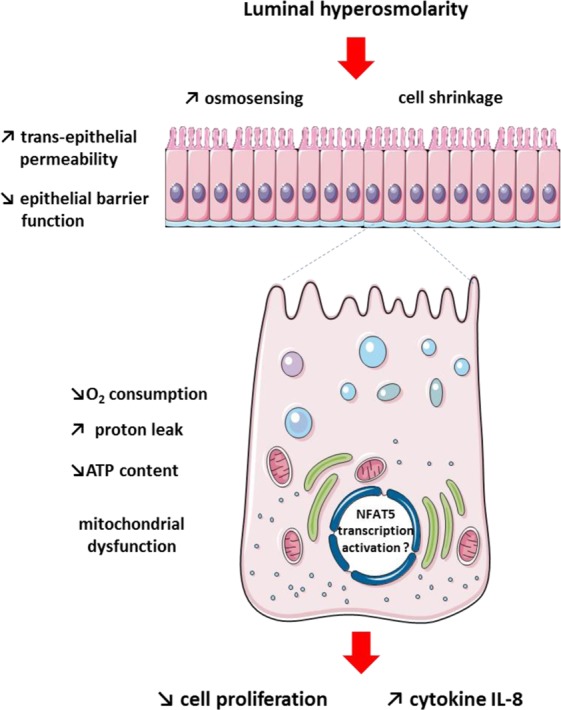
Schematic view of the intestinal epithelial cell response to luminal hyperosmolar environment. Luminal hyperosmolarity is responsible for mitochondrial dysfunctions in colonocytes that are characterized by decreased oxygen consumption and increased proton leak resulting in altered mitochondrial ATP production with a consequent reduction of the ATP intracellular content. This coincides with a slowdown of colonocyte proliferation, alteration of epithelial barrier function and increased release of the pro-inflammatory cytokine IL-8 by colonocytes. The cell shrinkage provoked by increased luminal osmolarity results in an osmoadaptive cell response which is likely to limit the deleterious effects of luminal hyperosmolarity.

Our study indicates that hyperosmotic environment, although not affecting the viability and adhesion of colonocytes, decreases the mitochondrial oxygen consumption and cell proliferation with a concomitant reduction of the ATP cell content (Fig. 5). These results coincide with a marked loss of epithelial barrier function together with an increased secretion of the pro-inflammatory IL-8 in Caco-2 monolayer. The modified expression of genes related to energy metabolism in both proliferative and differentiated colonocytes after 24-h treatment (but not after 2-h) suggests an adaptive process in response to mitochondrial activity alteration. Regarding the expression of genes encoding protein involved in TJ structure and electrolyte transport, our findings indicate a rapid cell response to hyperosmotic stress probably via NF-AT5, followed by a return to basal gene expression after 24-h. These patterns of responses, that are concomitant with a dynamic regulation of paracellular and trans-epithelial permeabilities in order to restore more favorable osmotic condition, would favour a return to normal epithelial function.

It is worth noting that the data presented here were obtained *in vitro* using a simplified model of human epithelial colonic cells, thus in conditions obviously different that the one characterizing the *in vivo* situations (e.g. protection of cells by mucous layers, organization in colonic crypts). With these reservations in mind, our data suggest that hyperosmotic aggression of the colonic epithelial cells, affects their energy metabolism, proliferation, ATP cell content and cell barrier integrity. Such findings are worth to be taken into account in pathological situations such as IBD, since osmotic stress is likely one player that affects intestinal epithelial cell homeostasis in the course of intestinal mucosal inflammation^15^.

## Materials and Methods

### Caco-2 cell culture

The human colon adenocarcinoma cell line Caco-2, used between passages 45 and 65 were cultured at 37 °C under 5% CO_2_ atmosphere in Dulbecco’s modified Eagle’s medium (DMEM) containing 4 mM L-glutamine and 1 mM sodium pyruvate and supplemented with 15% (v/v) heat inactivated fetal bovine serum and 0.1 mM non-essential amino acids. We used pre-confluent cells (between 3 or 7 days after seeding depending on experiments) as “proliferative” or “undifferentiated” cells, and cells obtained from monolayers 15 days after seeding on porous filters (characterized by a TER > 300 ohm/cm^2^) as “differentiated” cells. Table 3 summarizes the cell stages and the time points for the tests performed. For testing fluorescent marker permeability, the growth medium used was without phenol red. After treatment with the different hyperosmotic media, the cells were observed using a light microscope.

**Table 3:** Summary of experimental protocols.

	Proliferative cells (3 to 7 days after cell seeding)	Differentiated cells (15 days after cell seeding)
**2-h tests**:	Cell viability and proliferation	Gene expression
	Oxygen consumption	TER and markers of epithelial permeability
**24-h tests**:	Oxygen consumption	Gene expressions
	Mitochondrial gene expressions	TER and markers of epithelial permeability
	IL8 secretion	IL8 secretion

Proliferative Caco-2 cells (3 or 7 days post-seeding on plates or porous filters) were used before confluence. Differentiated Caco-2 monolayers (15 days post-seeding on porous filters) had a TER > 300 ohm*cm^2^.

### Hyperosmotic stress

Cell culture medium, with an osmolarity equal to 336 mOsm/L, was used for normal osmotic condition (control). In this latter medium, 30 mg/mL or 60 mg/mL mannitol were added to obtain 500 mOsm/L (for 50% intermediate hyperosmotic stress) or 680 mOsm/L (for 100% high hyperosmotic stress) respectively. Osmolarity was checked with an automatic micro-osmometer (Roebling).

### Viability tests

Cell viability after hyperosmotic stress was assessed by different methods: i. by counting the adhering cells obtained after trypsinisation and estimating their viability using the trypan blue method; ii. by measuring the LDH activity released in the apical medium by necrotic cells and that contained in the attached cells after treatment with 0.1% triton using the TOX7 kit (Sigma), then calculating the ratio of viable cells on total cells; iii. by measuring the mitochondria-dependent reduction of MTT (0.5 mg/mL) to formazan after 60 min incubation at 37 °C and reading of the optical density at 570 nm; and iv. by measuring the ATP cell content in adhering cells using the luminescence-based ADP/ATP ratio assay kit (Sigma). The viability tests were performed on cells grown for 3 days before hyperosmotic stress assays.

### Epithelial barrier function

Epithelial barrier function test was performed using Caco-2 cells grown as a monolayer on 12 mm Transwell^®^ filters and differentiated during 15 days post-seeding at 1.12 × 10^5^ cells per filters in the same cell Caco-2 medium with 1% penicillin-streptomycin. The following parameters were measured: i. the TER (in ohm × cm^2^) using an epithelial ohmmeter (Word Precision Instrument EVOM, Sarasota USA) and ii. the epithelial permeability to macromolecules after exposure to 62.5 μM FITC-dextran 4000 Da (FD4, Sigma) and to 2.7 μM of HRP (type II, Sigma) added at the cell apical side. At the end of the hyperosmotic stress, the amount of FD4 flowed at basal side was determined measuring the fluorescence in the Infinite^®^ 200 Pro spectrofluorimeter (TECAN, Switzerland) with an excitation and emission wavelengths of 490 nm and 520 nm respectively and quantified against a standard curve. For the measurement of HRP transport through the Caco-2 monolayer, HRP activity in the cell basal media of each sample was assessed using 0.003% H_2_O_2_ as substrate and 0.080 mg/mL o-dianisidine as dye in 0.01 M sodium phosphate, pH 6.0; after 10 minutes of reaction, that was stopped with 0.03% sodium azide, the sample absorbance was measured at 460 nm and plotted against a standard curve. The epithelial permeability towards the different compounds was expressed as pmol/mL.

### Ussing chamber experiments

Caco-2 cells were grown as a monolayer on 12 mm Snapwell^®^ filters and differentiated during 15 days post-seeding in the same Caco-2 cell culture medium as indicated above. Before performing Ussing chamber experiments, Caco-2 monolayers were treated during 22-h on the apical side with isosmotic, 50% hyperosmotic or 100% hyperosmotic media. Then each filter was mounted in the EasyMount Ussing chambers (Physiologic Instrument Inc, San Diego, CA) and bathed on the apical side in the same experimental media during additional 2-h to reach an overall 24-h treatment. The dual-channel epithelial voltage clamp EC825A (Warner Instruments LLC, USA) was used to record the monolayers *V*_t_ difference (in mV), that is mainly generated by the activity of the basolateral membrane Na^+^/K^+^-ATPase pump, and represents the driving force to the Na^+^-coupled apical transport. By clamping the monolayer to 0-mV, we obtained the *I*_sc_ (in μA/cm^2^) which reflects the sum of the active ionic fluxes across the monolayer and thus the net result of its absorptive and secretory capacity.

### Mitochondrial metabolism

8 × 10^4^ Caco-2 cells were seeded in 25 cm^2^ flasks and grown 7 days before recovery by trypsine (0.25 g/L) in phosphate-buffered saline (PBS) containing 1 g/L EDTA. Approximatively 5 × 10^6^ cells in 2 mL of an air saturated respiration medium (in mM: 20 Hepes, 200 mannitol, 5 KH_2_PO_4_, 2.5 MgCl_2_, and 0.5 EGTA, pH 7.4, enriched with 0.1% bovine serum albumin) were placed in the oxygraph chamber for oxygen consumption measurement rate. Cell respiration was recorded in real time with an “O2k”Oroboros apparatus (Innsbruck, Austria) at 37 °C. Oxygen consumption rates were obtained directly from the Datalab 4 software and were calculated as the negative time derivative of oxygen concentration in the closed respirometry chamber. The respiratory fluxes were corrected automatically for instrumental background by Datalab taking into account the oxygen consumption of the oxygen sensor and oxygen diffusion out of or into the oxygraph^61^.

For intact cells, after stabilization of the ST3, that was measured in the absence of any exogenous agent, and is considered as the 100% reference value, the inhibitor of mitochondrial ATP synthase (complex V, CV) oligomycin was added at 0.5 µg/mL concentration. This allows measuring the proton leakage through the inner mitochondrial membrane. When the oxygen flux was stable, FCCP was added at 1.5 µg/mL concentration allowing the measurement of the cell maximal respiratory capacity. OXPHOS rate was determined in the presence of 1.5 mM saturating ADP concentration and complex I and II substrates using cells firstly permeabilized by adding digitonin (50 µg per 5 × 10^6^ cells).

### Gene expression

Gene expression after hyperosmotic stress was investigated by quantitative RT-PCR on Caco-2 cells grown for 7 days on 6-well plates (with 1.6 × 10^5^ cells per well at seeding) or 15 days on 24 mm Transwell^®^ filters (with 4.67 × 10^5^ cells per filter at seeding). After performing the hyperosmotic stress test, media were removed and the cells were washed twice with PBS. Then, the cells were scraped in RLT/β-mercaptoethanol cell lysis buffer (RNAeasy^®^ mini kit, Qiagen) and total RNA was purified following the manufacturer protocol including an RNase-free DNAse I (Qiagen) treatment. One μg of total RNA was reverse-transcribed with 50 U of MultiScribe Reverse Transcriptase following the instruction of the High Capacity cDNA Reverse Transcription kit (Applied Biosystems™), and 25 ng of cDNAs were used in the PCR reactions with the Fast SYBR Green Master Mix (Applied Biosystems™). We tested the expression level of some genes related to tight junction, mitochondrial metabolism, electrolyte transport and inflammation (Table 4). The hypoxanthine-guanine phosphoribosyltransferase (*HPRT*) and the 60S ribosomal protein L18 (*RPL18*) genes were amplified and used as reference genes. Relative gene expression was calculated with the 2^−ΔΔCt^ method. Gene and protein names given in this article are in accordance with the HUGO Gene Nomenclature Committee (https://www.genenames.org/) and UniProt (https://www.uniprot.org/) respectively.

**Table 4:** List of primers used in gene expression studies.

Protein name (*Gene symbol*)	Upper primer (5′ → 3′)	Lower primer (5′ → 3′)
Occludin (*OCLN*)	ACCCCCATCTGACTATGTG	CTTGCTCTGTTCTCTTTGACC
F11 receptor (*JAMA*)	CACCACCAGACTCGTTTG	GCCTTCCTCAGAGACCATAC
Zona occludens Protein 1 (*TJP1*)	CCGTGTTGTGGATACCTTG	GCCTGCTGTTTTTGGAG
Claudin-1 (*CLDN1*)	TGAGGATGGCTGTCATTG	GGTAAGAGGTTGTTTTTCGG
Claudin-2 (*CLDN2*)	AGCAGCCCAGACAATGAG	TAGGATGTAGCCCACAAGTTG
Claudin-5 (*CLDN5*)	TGGGTCACTGGGAACTTC	AGTCTCTGGCAAAAAGCG
Na^+^/K^+^-ATPase alpha subunit (*ATP1A1*)	GGAGACGAGGAACATTGC	GACACACCCAGGAACACAG
Na^+^/H^+^ type 1 exchanger (*NHE1*)	GGTTCTGGCTGTCTTTGAG	ATGTGGGAGGTAAATCGG
Na^+^/H^+^ type 3 exchanger (*NHE3*)	TGACGCTGGTCTTCATCTC	GTGCTCGCTCCTCTTCAC
Na^+^/K^+^/2Cl^−^ type 1 co-transporter (*NKCC1*)	TACCCACACCAACACCTACTAC	CACGACTCCTTTACTTTCTGC
Aquaporin 3 (*AQP3*)	CTTCTTGGGTGCTGGAATAG	TGCCTATGAACTGGTCAAAG
ATP synthase (*ATP5F1B*)	CATCTCCTTCGCCAAAAG	TGATTCTGCCCAAAGTCTC
Cytochrome C oxidase 5A (*COX5*)	CGCTGGGTAACATACTTCAAC	GATGACATAGGGGTAGATTTCC
Ubiquinol cytochrome C reductase binding protein (*UQCRB*)	AAGGCAACGCTTCTCTTTC	CCCCAGTTTATTGAATCCTG
NADH Ubiquinone oxidoreductase S1 (*NDUFS1*)	GCAGGAGTAGATGATTTGGG	GGCATAGGGCTTAGAGGTTAG
Succinate deshydrogenase (*SDHD*)	ACCGACCTATCCCAGAATG	CTGAAAGTGCCAAAAGCC
Citrate synthase (*CS*)	TTGGCTGACCTGATACCTAAG	CAAGATACCTGTTCCTCTGTTG
Cyclooxygenase 2 (*PTGS2*)	TCAGCCATACAGCAAATCC	GGTGTTGAGCAGTTTTCTCC
Nuclear factor in activated T cell 5 (*NFAT5*)	GGACATTGAAGGCACTACTG	TTGGAGAAGAGGGTGTTTG
Interleukin 6 (*IL6*)	CTGAACCTTCCAAAGATGGC	AGCAGGCTGGCATTTGTGGT
Interleukin 8 (*CXCL8*)	TCTCAGCCCTCTTCAAAAACTTCTC	ATGACTTCCAAGCTGGCCGTGGCT
Hypoxanthine Phosphoribosyltransferase 1 (*HPRT*)	ATGTTGCGTTGGAAGTGTAG	AGGTATGCGGTATTTGGC
60S ribosomal protein L18 (*RPL18*)	ACCTGGCCGAGCAGGAG	TGGAGTTGGTTCTTCTGGC

### Cytokine release

IL-8 secretion after hyperosmotic stress was measured in Caco-2 cells plated on 6-well plates and grown for 3 days; and in differentiated Caco-2 cells initially plated on 24 mm Transwell^®^ filters and grown for 15 days before apical hyperosmotic stress. IL-8 cytokine released from cells in the culture media after hyperosmotic stress was detected using the Human IL-8 ELISA Ready-SET-Go!^®^ kit (Invitrogen) following manufacturer’s instructions.

### Statistical analysis

Values are expressed as means ± standard errors of the mean (s.e.m.). The effect of treatment was analyzed with GraphPad Prism 8.1.1 software using a two-way analysis of variance (osmotic stress (50% or 100% hyperosmotic media) and time (2-h or 24-h treatment) or cell stage (proliferative-undifferentiated or differentiated)). Bonferroni’s posthoc tests were used for pairwise comparison. Differences were considered statistically significant at *P* < 0.05.

## Data Availability

The datasets generated and analysed during the current study are available from the corresponding author on reasonable request.
